# Considerations for an Access-Centered Design of the Fever Thermometer in Low-Resource Settings: A Literature Review

**DOI:** 10.2196/humanfactors.6778

**Published:** 2017-01-18

**Authors:** Rikako Iwamoto, Ana Laura Rodrigues Santos, Niels Chavannes, Ria Reis, Jan Carel Diehl

**Affiliations:** ^1^ Design for Sustainability Industrial Design Engineering Delft University of Technology Delft Netherlands; ^2^Department of Public Health and Primary Care Leiden University Medical Center Leiden University Leiden Netherlands; ^3^ Amsterdam Institute for Social Science Research Faculty of Social and Behavioural Sciences University of Amsterdam Amsterdam Netherlands; ^4^ The Children's Institute School of Child and Adolescent Health University of Cape Town Cape Town South Africa

**Keywords:** fever diagnostics, sub-Saharan Africa, thermometer, low-resource settings, design, patient journey, product-service system

## Abstract

**Background:**

The lack of adequate information about fever in low-resource settings, its unreliable self-assessment, and poor diagnostic practices may result in delayed care and under-or-overdiagnosis of diseases such as malaria. The mismatches of existing fever thermometers in the context of use imply that the diagnostic tools and connected services need to be studied further to address the challenges of fever-related illnesses and their diagnostics.

**Objective:**

This study aims to inform a product-service system approach to design a reliable and accessible fever thermometer and connected services, as well as contribute to the identification of innovative opportunities to improve health care in low-resource settings.

**Methods:**

To determine what factors impede febrile people seeking health care to access adequate fever diagnostics, a literature search was conducted in Google Scholar and PubMed with relevant keywords. Next, these factors were combined with a patient journey model to design a new product-service system for fever diagnostics in low-resource settings.

**Results:**

In total, 37 articles were reviewed. The five *A*s framework was used to categorize the identified barriers. The results indicate that there is a poor distribution of reliable fever diagnostic practices among remote communities. This paper speaks to the global public health and design communities. Three complementary considerations are discussed that support the idea of a more holistic approach to the design of fever diagnostics: (1) understanding of the fever diagnostics patient journey, (2) identifying user groups of the thermometers in a specific health care system, and (3) assessing different needs and interests of the different users.

**Conclusions:**

Access to basic, primary health care may be enhanced with better information and technology design made through the involvement of system users.

## Introduction

In low-resource settings, fever-related illnesses and their diagnostics represent a particular challenge. Despite the improvements achieved through the Millennium Development Goals, more than 40% of the population of Africa, especially sub-Saharan Africa, lives in extreme poverty and suffers from high health care disparities [1]. While the disease burden of malaria, for example, is well quantified, the burden of other diseases is underappreciated. Existing literature refers to common misdiagnosis associated with a narrow vision of diseases; similarity of clinical profiles of illnesses; and lack of treatment guidelines, laboratory resources, and of adequate and complementary diagnostic tools [2-4]. Despite the successful adoption of rapid diagnostic tests, there are yet untapped opportunities to develop support tools to facilitate the distinction of often-neglected fever-related illnesses.

The fever thermometer is one of the simplest medical devices that are widely and commonly used to support almost all kinds of everyday health care in hospitals, health care centers, physicians’ offices, ambulances, and laboratories worldwide [5]. The threshold of fever differs significantly between different individuals [6]. This subjective nature is related to the fact that different variables influence the assessment of body temperature, such as age, gender, ethnicity, physical exercise, ambient temperature, body site of measurement, and operator techniques [7-29]. Common temperature assessment sites are oral, axillary, ear, and rectal and it is inappropriate to compare temperature readings measured at different body sites. The monitoring of fever enables caregivers to follow the course of an illness and evaluate the ability of the immune system to fight it. In addition, for hyperthermia and a group of high-risk illnesses (eg, heart problems and diabetes), fever can indicate a severe condition for which delayed treatment is not acceptable [30]. However, in low-resource settings, thermometers are not used nor understood by everyone [31,32]. For the majority of mothers and other caregivers of young children, tactile measurements of body temperature (eg, with a hand against the forehead) is often the only resource to assess fever [33-35]. Palpation performed by mothers is seen as a useful and accurate first step in deciding if and when a child of less than 5 years of age needs to be referred to health care services [36,37]. On the other hand, there is some divergence in the literature regarding the reliability and specificity of fever self-assessment and, consequently, the value of fever thermometers for the lower level of caregivers (ie, village health teams, including parents and community health workers). In fact, self-assessment has been shown to be inaccurate and unreliable when compared with the objective standard of rectal measurement of body temperature with a thermometer [31,38-40]. Introduction of a chemical thermometer (ie, forehead temperature strips), designed to be disposable after one-time use, made temperature recording easy and safe as an alternative method for assessment of fever in low-resource settings [41]. However, the sensitivity of the chemical thermometer is inaccurate and inconsistent and produces frequent false-positive results compared to the mercury thermometer [42]. Therefore, it is not recommended for use by health care providers [24,43]. This might indicate that the fever thermometer, as it is designed, may not entirely fulfill its purpose given its existing mismatch with the context and the end users of health care systems (ie, in sub-Saharan Africa). Poor diagnostics may lead patients to be overdiagnosed or diagnosed with the wrong disease, resulting in a waste of medical resources and contributing to resistance to medication. In addition, overlooked diagnosis may lead to inadequate and unnecessary self-treatment or neglected or delayed treatment of patients, which in turn brings related risks for the patient and their communities [4,44]. Thus, the mismatches of the existing medical devices in the context of use imply that the diagnostic tools and additional health care services need to be studied further in order to address the challenges of fever-related illnesses and their diagnostics and to fulfill users’ needs.

The objectives of this study are to inform a systemic (ie, design) approach to develop a reliable and accessible fever thermometer and connected services, as well as to contribute to the identification of innovative opportunities to improve health care in low-resource settings [45-47]. To address the challenge of fever diagnostics, it is of importance to comprehend the health care system and user contexts. This is achieved through a literature review to determine the factors preventing people from accessing and receiving adequate fever diagnostics and follow-up in low-resource settings. Next, these factors are looked upon from a systemic (ie, design) approach to propose complementary considerations for a product-service system approach for fever diagnostics. This could conclusively lead to maximizing the value in existing health care programs and health infrastructures and to improvements in the quality of health care services.

## Methods

### Search Strategy for the Literature Review

A literature review was conducted to identify the barriers to assessing body temperature in low-resource settings. In order to clarify and quantify the relationship between fever diagnostics and a health care system, Uganda was selected as a representative country of the sub-Saharan African region. Publications were retrieved from Google Scholar and PubMed using the following keywords: *fever* and *Uganda*, *barrier* and *febrile treatment*, *thermometer* and *diagnosis*, *drug shop*, *rural Uganda*, *healthcare*, *measuring body temperature*, and *misdiagnosis*. An additional keyword, *perception*, was used after retrieving the publications from the first search. Simultaneously, related articles were searched based on data extracted from citation indices. Articles were selected if they included qualitative and/or quantitative studies that identified barriers to assess the body temperature in resource-constrained environments, especially in Uganda. Articles were excluded if they only focused on specific countries in low-to-middle-income economies excluding Uganda.

### Study of Barrier Categorization

The five *A*s of access to care by Penchansky and Thomas [48,49] were used to categorize the barriers identified in the searched literature. Characteristics and expectations of both health care providers and their clients were grouped into the five *A*s: accessibility, availability, acceptability, affordability, and accommodation. This framework was selected among others for this study given its extensive use in the field of health care [48,50-53], its degree of detail, and its comprehensiveness regarding the different health care service *users*. Although the aim of this study was not to compare existing frameworks, the following description highlights the most relevant considerations for the authors’ choice.

In the five *A*s framework, accessibility refers to the geographic distribution of health care facilities. Availability relates the existing quantity of resources (ie, personnel and technology) with the ones required to meet the demands of the people. In the framework of Peters et al [50], for example, these two dimensions are merged and are therefore less adequate in circumstances where incomplete or unsuitable health care facilities are located nearby health care seekers. Affordability relates the direct and indirect charges related to health care services to the ability and willingness of health care seekers to pay them. Acceptability refers to the inherent characteristics of the system in place regarding genre, ethnicity, and social class, for example, and is often susceptible to mutual social and cultural appraisals. Finally, accommodation is determined by the extent to which the offered services are adjusted to match the client’s access capacity (eg, hours of operation and people's ability to receive treatment without prior appointments) [48]. This aspect, in particular, points toward an interesting service design component and the consideration that systems can purposefully be designed to adjust to the lifestyle of health care seekers that other frameworks do not include.

The two latter aspects are often merged into one dimension. In Prahalad’s innovations in the bottom of the pyramid [53], these two aspects combined are renamed *awareness* of providers. In Peters et al [50], acceptability is only described from health provider’s perspective. The 4 *As* framework of the World Health Organization [5] is focused on medical equipment. Accommodation and acceptability are described as technical *appropriateness* to context. In McIntyre et al [51] and in Grimes [52], access barriers to health care in low- and middle-income countries are categorized into only three dimensions: acceptability, affordability, and availability [51]. Accessibility and availability are merged and defined as “being at the right place, at the right time.” Accommodation and acceptability are seen as a corresponding dimension between a participant’s expectations and the services provided.

## Results

### Overview

A total of 37 articles were included and reviewed. These include 25 studies that relate to treatment of febrile illnesses, of which seven address fever diagnostics and three address health care services in Uganda. Also included in the literature were four studies that looked at medical devices in low-resource settings and two studies that addressed more generally the barriers to accessing health care in low-resource settings. We identified 11 main barriers to accessing and receiving adequate fever diagnostics that were divided into the five categories (see Table 1) [3,4,31,33,40,44,54-63]. They will be discussed in detail in the following sections.

### Accessibility of Health Care Services in Uganda

The difficulty and delay in accessing treatment of febrile illnesses is attributed to a large extent to the physical distance between health care providers and health care seekers. The physical distance to health care providers influences people’s choices of health care providers when seeking care for febrile illnesses. This mostly affects people living in rural areas in Uganda, where the majority of the population (84.4%) lives [54]. According to the definition from the Ugandan government, the health care sector can be divided into the public sector and private sector [56] (see Textboxes 1 and 2).

**Table 1:** Barriers to access of diagnostics of fever-related illness.

Category	Barrier	Reference
Accessibility	Distribution of, and distance to, health care providers	[4,44,54-56]
Availability	Incomplete medical infrastructure	[3,40,56-60]
	Failure to utilize medical equipment	[59,60]
	Lack of health care professionals	[61]
	Lack of training for health care professionals	[3,55,60,62]
	Poor supervision by local authorities	[54,56]
Acceptability	Cultural beliefs and influence from community members	[58]
Accommodation	Mismatch between available information and awareness, knowledge, and education needs	[57,63]
	Lack of relevant and complete diagnostic information	[31,33]
Affordability	Cost of treatment	[54-57]
	Cost of transport to health care provider	[56,57]

**Types of public health care providers in Uganda [ 56].**

Public health care providers:

- National referral hospital (ie, advanced tertiary care)
- Regional referral hospital (ie, specialists services)
- General hospital (ie, general hospital care, secondary services, laboratory, and x-ray)
- Health center IV (ie, outpatients, wards, theater, laboratory, and blood transfusion)
- Health center III (ie, outpatient services, maternity, general ward, and laboratory)
- Health center II (ie, outpatient services only)
- Health center I (ie, outpost for outreach services)

**Types of private health care providers in Uganda [ 56].**

Private nonprofit health care providers:

- Nongovernmental facilities

Private for-profit health care providers:

- Medical clinics
- Dental clinics
- Drug shops
- Maternity homes

Private informal health care providers:

- General merchandise shops
- Traditional healers
- Mobile health care providers
- Unqualified persons

**Figure 1:**
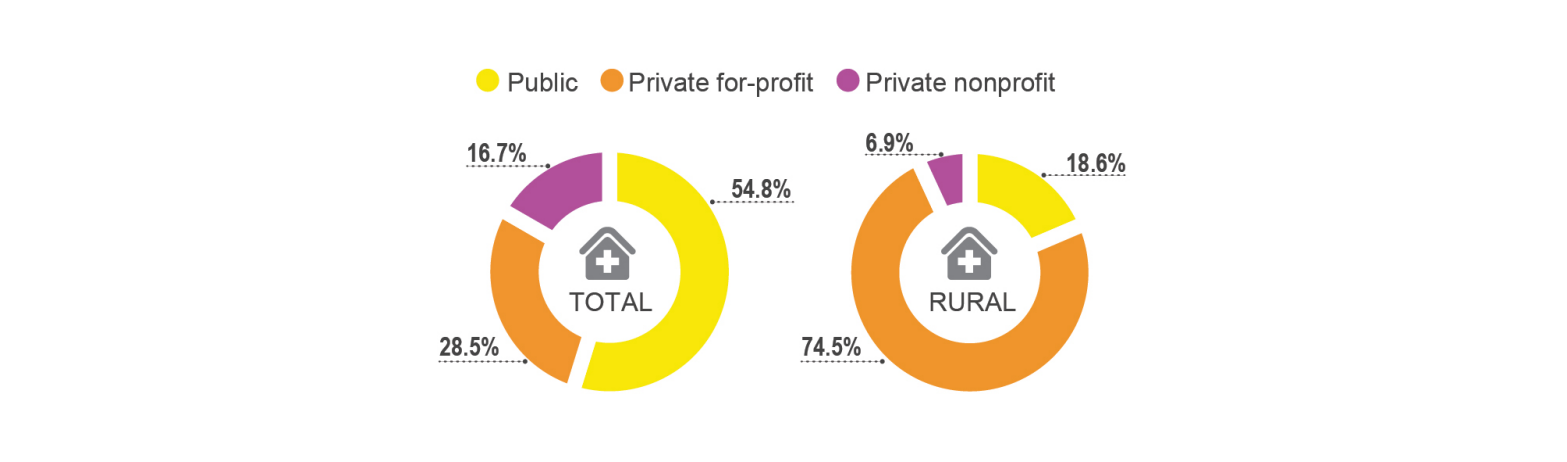
Share of health care facilities in all of Uganda (total) and in rural Uganda [56].

The private health sector is categorized into private for-profit, private nonprofit, and informal providers. Drug shops categorized into private for-profit account for the largest proportion of all facilities in the private health sector in all districts except Kampala, where more clinics than drug shops can be found [56]. Public facilities, which include hospitals and health centers (II, III, and IV), make up 54.8% of the total Ugandan health care facilities, while 28.5% are private for-profit and 16.7% are private nonprofit [64] (see Figure 1). However, the distribution of health care facilities in rural areas is significantly different from the distribution of health care facilities in Uganda as a whole. While more than half of all health care providers in Uganda are from the public sector, the public sector accounts for only 18.6% in rural districts where the majority of health care providers are private for-profit (74.5%) (see Figure 1).

**Figure 2:**
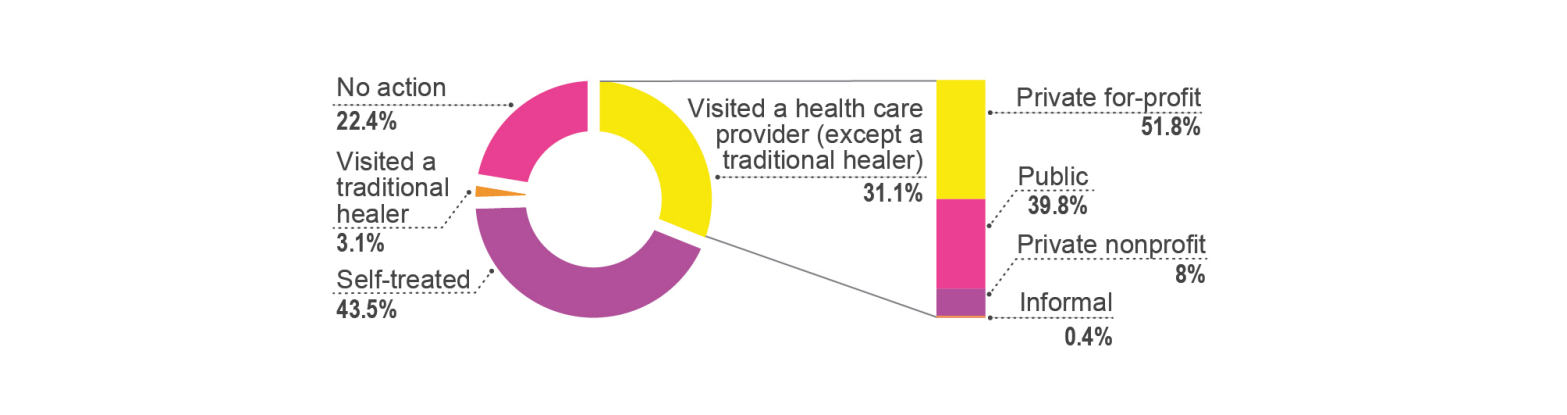
Distribution of health care received by people with febrile symptoms [56].

**Figure 3:**
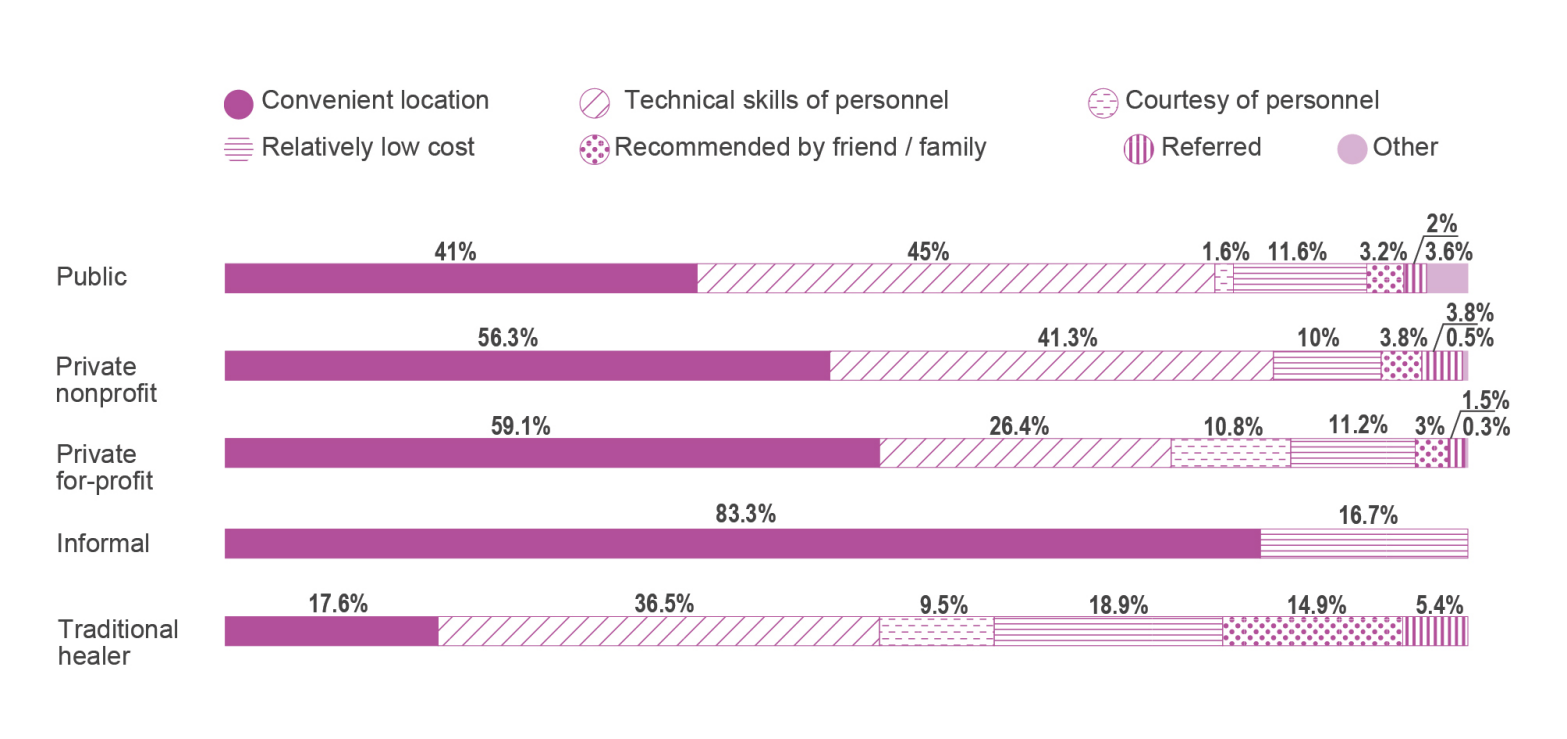
Reasons why caretakers chose specific health care providers for fever treatment [56].

Regarding the distribution of care sought by people with febrile symptoms, 31.1% of people sought care from a health care provider. Among health providers, excluding traditional healers, the main providers visited by people with febrile symptoms were private for-profit providers (51.8%), followed by public sector (39.8%) and private nonprofit providers (8%) (see Figure 2). Despite other treatment options being available in the community, the majority of people suffering from febrile symptoms treated their febrile illnesses by themselves at home (43.5%) or took no action (22.4%) [56]. The main reason given for visiting private providers instead of public health care providers is the convenience of location (ie, proximity) (see Figure 3). This may be explained by the fact that the great majority of health care facilities are private for-profit (74.5%) in rural Ugandan districts (see Figure 1). The distance to health care facilities also impacts the timing of care. Delay of treatment for fever occurred less among the people who perceived the distance between their home and the health care provider to be less than 1 km compared to those who perceived it to be more than 1 km [44].

### Availability of Professional and Well-Resourced Services

**Figure 4:**
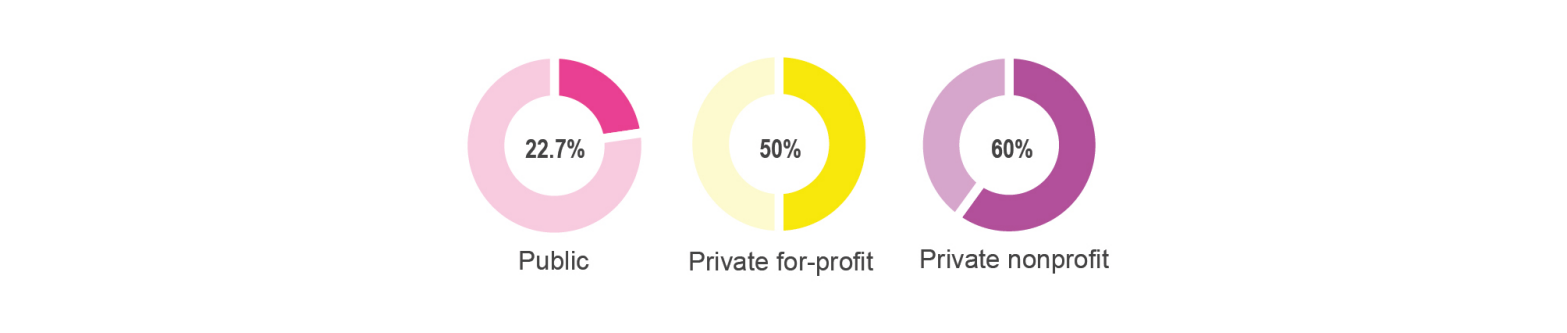
Availability of thermometers at different health care provider facilities [56].

Among the health care facilities, public facilities are perceived as having qualified and experienced health care providers by people seeking care for fever [55]. However, the government health sector is underresourced and understaffed and primary diagnostic equipment is frequently missing (see Figure 4). Compared to the private sector, where more than half of the private for-profit and nonprofit facilities are equipped with thermometers, public facilities were the worse equipped with thermometers among the formal health care facilities [56]. Furthermore, even though thermometers are available, a chronic understaffing problem in the public sector leads clinicians to routinely and inadequately assess patients’ body temperature by placing a hand on their foreheads, versus utilizing thermometers, when it is peak time in the waiting room [57].

**Figure 5:**
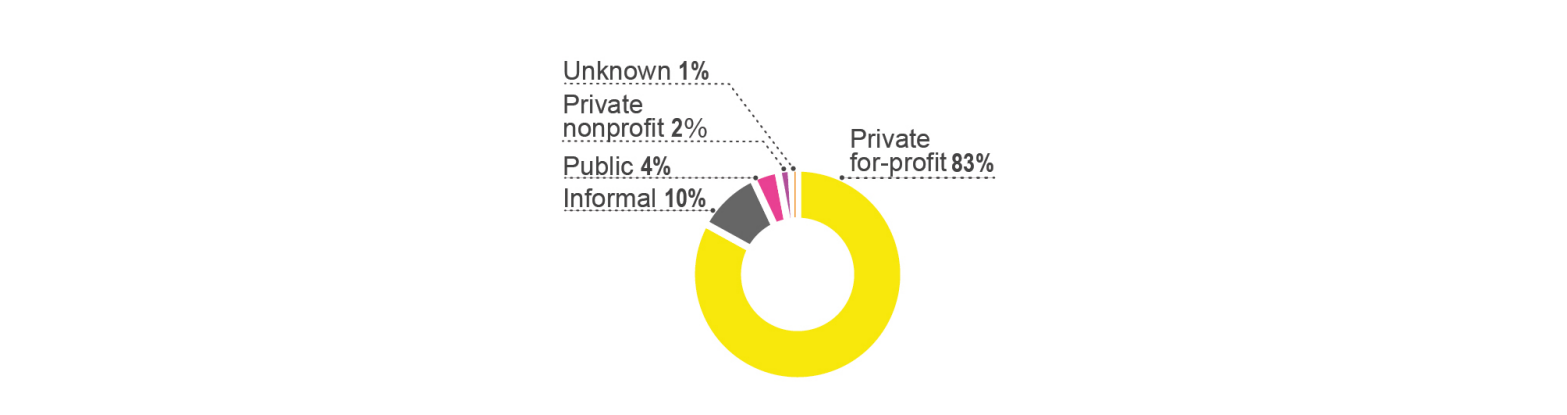
The distribution of health care facilities where medicine was purchased [56].

Regarding staff qualification, the private sector is invariably inferior to the public sector [3]. However, even though health care providers in the public sector were perceived as experienced, only 3 in 10 public health care professionals were able to diagnose 4 out of 5 very common illnesses (ie, malaria with anemia, acute diarrhea with severe dehydration, pneumonia, pulmonary tuberculosis, and diabetes mellitus). Among the most common, malaria with anemia was the least likely to be diagnosed correctly and only 9% of the cases were recommended the appropriate treatment [3]. Fever is more likely to be assessed by tactile measurement (ie, placing the palm or back of the hand on the forehead) than with a fever thermometer and the changes of fever over time are observed by patients or parents of child patients [31]. The absence of a fever thermometer at home hampers and delays the treatment of fever. Clinicians and nurses claim that people should have thermometers ready at home to quickly and objectively assess body temperature and be able to deduce how serious an illness may be [57]. Due to medical resource constrains in the public sector, the private for-profit facilities perform a key role in the supply of medicine. The main providers (83%), where febrile patients purchase medicine, are the private for-profit facilities and the second-major provider (10%) is the informal sector (see Figure 5) [56]. Informal providers are numerous, nearby, and more consumer oriented [65]. However, the private sector’s knowledge and quality of treatment at drug shops are recognizably limited [4]. While personnel with good technical skills was the main reason given for choosing public providers (45%), 26.4% of people perceived that the private for-profit providers had personnel with good technical skills (see Figure 3). In addition, even though 85% of the public health care facilities were inspected by local authorities monthly or quarterly, only half of the private for-profit facilities (54%) were inspected monthly or quarterly and 36% were never inspected at all [56].

### Acceptability of Existing Health Care Services

Besides the associated cultural beliefs regarding the subjectivity of fever itself, there are relevant acceptability aspects about how and when fever is measured among community social networks. Social networks and common practices in the communities play an important role in fever-related health care decisions. Nsungwa-Sabiiti et al [66] describe how mothers are often reluctant to seek professional health care for their ill children at an early stage [58]. Feeling unwell with any kind of fever symptom is perceived as the most important disease in their community and is believed to be caused by something you ate or drank, environmental conditions, mosquitoes, and being a symptom of other diseases. There was consensus among the members of the community that care for febrile symptoms is to be sought from the informal sector before visiting the formal sector. The health care facilities are visited as a definitive way to care for febrile people after treatment with herbs and medicine purchased from the shops [67].

### Accommodation of Technologies and Services to Existing Needs

Technologies and services do not accommodate the needs, expectations, or habits of health care seekers in several ways. First, the reading of the thermometer, as it is designed, is often not understood. This may be due to the multiplicity of different meanings people associate with fever or a febrile condition. In a setting where there is little or poor information available about the required follow-up of fever with regard to required dosages of medication and risks associated with diseases, the diagnostic information provided by the thermometer does not match the semantics associated with fever [57,66]. Second, this is not limited to households. The lack of knowledge in health care services to manage nonmalaria febrile illnesses results in health workers treating patients that have a negative malaria test result with antimalarial medicines [63]. As the patients expect to receive care for their symptoms, it is essential to provide appropriate management and information of febrile symptoms to those people who do not have a malaria infection. Finally, another limitation related to the diagnostic information provided by most fever thermometers is related to fever kinetics. The reporting of patients’ fever kinetics (ie, the progress of fever over time versus a punctual measurement) is essential for an adequate and accurate diagnosis. Since people often seek health care with delay, it is important for health care providers to know if the progress of fever based on memory is reliable [31]. Despite the accuracy of digital fever thermometers, since normal body temperature has individual variations, patients and parents need to know the patients’ baseline morning temperature to be able to judge an increased temperature as fever [7].

### Affordability

**Figure 6:**
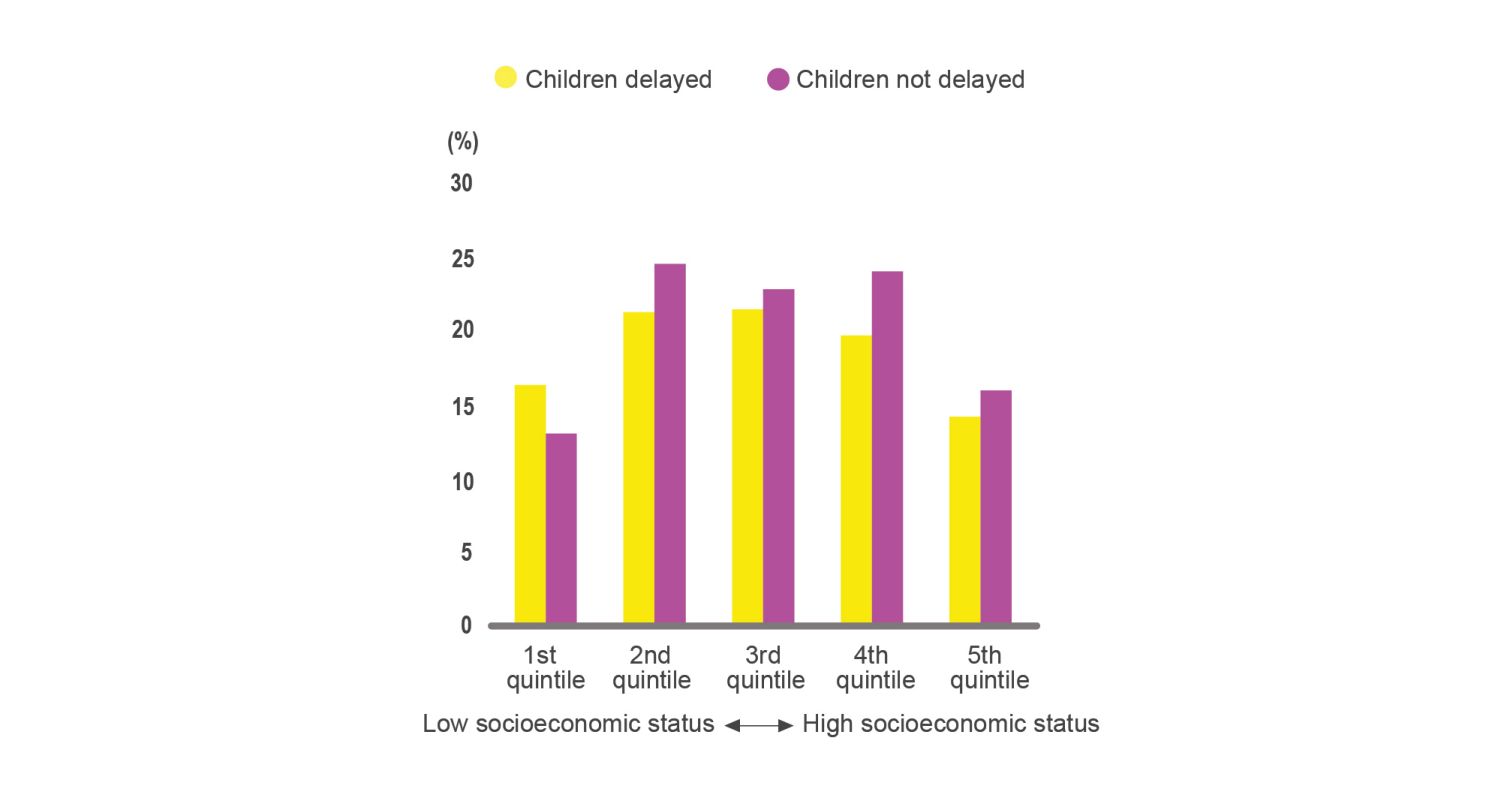
Percentage of febrile children taken outside of their home for care within 24 hours (not delayed) versus after 24 hours (delayed) in different socioeconomic quintiles [44].

**Figure 7:**
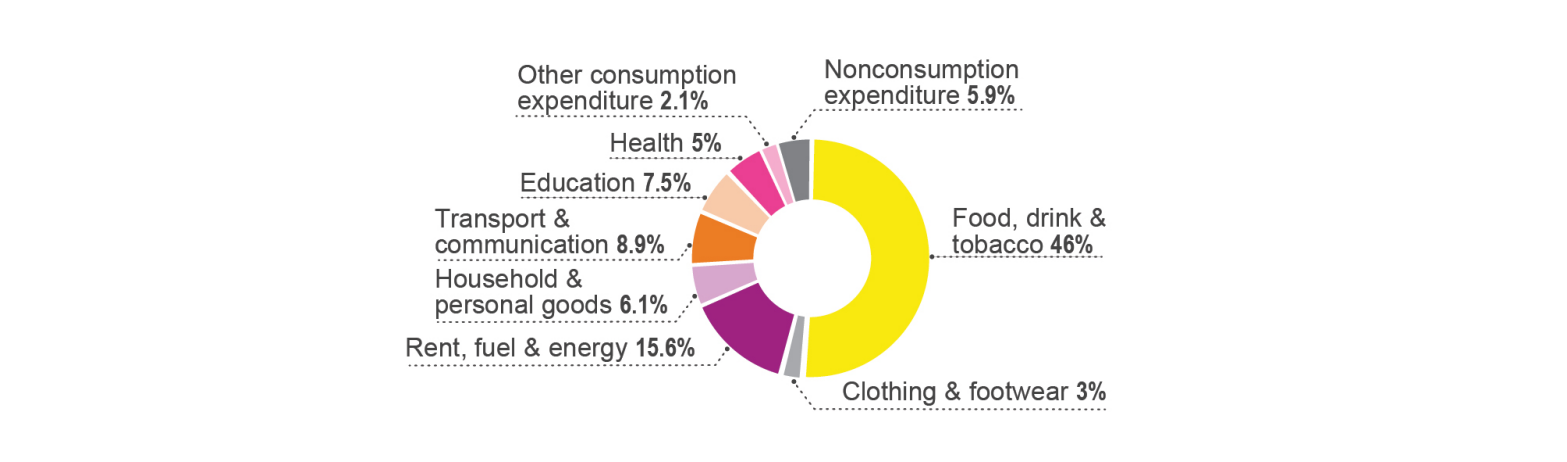
Share of household expenditure by item group (% of total expenditure) [64].

While the cost of treatment was a relatively minor determinant among a range of barriers to assess primary fever diagnosis compared to accessibility, the financial challenge is still one of the critical barriers and a concern for people seeking treatment for fever. The socioeconomic status of households has an effect on the timing of care seeking. Figure 6 shows the percentage of febrile children in Uganda that effectively sought timely care in different socioeconomic quintiles. Children with the lowest socioeconomic status were more likely to receive delayed care [44]. Although public health care services in Uganda are meant to be free, most patients have to pay for the treatment they receive and for the costs implied in transportation. Konde-Lule et al [56] demonstrated that in the public sector, half of the clients were charged for health services and paid an average of UGX 5381 (about €1.4). As expected, the majority of clients seeking care in private for-profit (86.5%) and nonprofit (84%) facilities were charged. The lowest average amount for health services is in private for-profit facilities where they cost UGX 4626 (about €1.2); the highest average amount for health services is in private nonprofit facilities where they cost UGX 7647 (about €2). The average monthly income of employees in 2013 was UGX 491,000 (about €128). Figure 7 illustrates the share of household expenditure by item group; health care expenditure accounts for 5% of total household expenditures [64].

## Discussion

### Considerations for a Fever Diagnostics Product-Service System Design

#### Overview

This study is aimed at obtaining a comprehensive picture of the context surrounding patients and people seeking fever diagnostics in low-resource settings in order to inform a product-service system design approach and biomedical engineering approaches to fever diagnostics [68]. The field of medical devices and diagnostics design for low-resource settings is recent but broad. Literature about the field comes from contributing disciplines such as management science, technology transfer, industrial design, user-centered design, ergonomics, and biomedical engineering. Literature frequently refers to the current misfit of medical devices in the context of use [69-74] and models or frameworks for improved design processes [75-77]. In this paper, the authors argue that a systemic (design) approach may be more suitable to address fever diagnostics in low-resource settings by creating meaning and value to end users through not only new technologies, but also new services or processes. A user-centered design perspective, where user tasks are closely observed, runs the potential risk of placing a single aspect of use and interaction in isolation [78,79] because most people within a health care system are involved with two or more primary participants: consumers, patients, clinicians, and technicians. Health care is a very large social system and involves many participants and roles in addressing the recovery of individual and social health. Therefore, a product-service system approach [80,81] to fever diagnostics could contribute to the enhancement of the quality of health services. This is true because it considers the physical and sociocultural environments; the financial, organizational, and scientific concerns of the health care systems; resource availability; users’ level of knowledge; and the industrial and economic realm of medical devices [60,82,83].

The authors propose three complementary considerations for product-service systems design of fever diagnostics: (1) the fever diagnostic patient journey to clarify the situations in which health seekers encounter barriers, (2) the different users of a fever thermometer across that journey, and (3) the different capabilities and needs of the users.

In the next sections, we will discuss these considerations in connection to the barriers to fever diagnostics as identified in the literature review.

#### Understanding the Fever Diagnostics Journey

A patient’s (and health professional’s) journey helps to identify and understand the context in which interactions between thermometers and users occur and to identify when patients experience difficulties in accessing fever diagnostics in the health care system. Since body temperatures can be taken in different situations (eg, health clinic, hospital, and household), it is important to obtain a contextual picture of users and their user tasks. In addition, it widens the scope of analysis of fever diagnostics and contributes to the identification of innovation opportunities not only by means of products (ie, fever thermometer), but also services and programs. The authors categorized the barriers into a fever diagnostics journey model (see Table 2). The model was created by combining Table 1 (barriers to access) with the patient journey model proposed by Manchaiah and Stephens [84], focusing on the three phases related to access, namely *awareness*, *movement*, and *diagnostics*. Table 2 relates the barriers identified in the literature with a set of steps in fever diagnostics. In the table, some barriers are associated with awareness, others with movement or decision-making, and others with the diagnostic itself. Awareness barriers are related to the perceptions and habits regarding fever as well as the lack of appropriate information about the symptoms, why fever should be measured, what should be measured, and how and when to measure fever. In regard to the decision of whether to look for treatment, barriers include the difficulty of access to health care services and their associated costs. Finally, diagnostic barriers are related to the infrastructure available in terms of technology and human resources. This division of barriers provides a clear picture of how fever diagnostics can be addressed in a holistic way to identify opportunities for innovation that focus beyond the fever thermometer. This includes the design of meaningful displays and information, easy algorithms for decision-making, and connected services.

**Table 2:** Barriers for assessing body temperature throughout the fever diagnostics journey.

Category	Phases and their associated barriers		
	Awareness	Movement	Diagnostics
Acceptability	Cultural beliefs and influence from community members	N/A^a^	N/A
Accessibility	Mismatch between available information and awareness, knowledge, and education needs	Distribution of, and distance to, health care providers	N/A
Availability	N/A	N/A	Incomplete medical infrastructure Failure to utilize medical equipment Lack of health care professionals Lack of training of health care professionals Poor supervision of health care facilities by local authorities
Accommodation	N/A	Lack of relevant and complete diagnostic information	N/A
Affordability	N/A	Cost of treatment Cost of transport	Cost of treatment

^a^N/A: not applicable.

#### User Groups of Fever Thermometers

**Figure 8:**
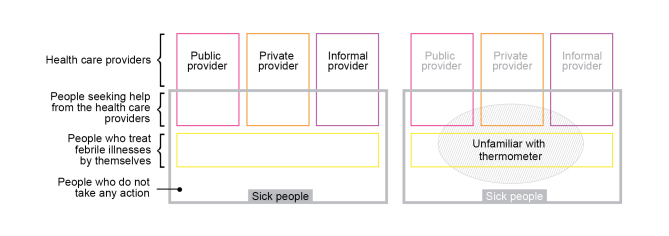
User groups of thermometers (left) and people who are unfamiliar with thermometers (right). Image is not proportional.

Figure 8 provides a map of thermometer user groups involved in fever diagnostics in Uganda. In the diagram, there are two general types of roles: health care providers and sick people with fever symptoms. Three different types of health care providers are identified: public, private, and informal facilities. There are three types of sick people: those who seek help from the health care providers, those who are aware of the necessity to enhance their health condition and treat febrile illnesses by themselves, and people who do not take any action. In addition, it could be assumed that there are two types of febrile patients: those who are familiar with using thermometers and those who are unfamiliar. The analysis in this study reveals that each group of users deals with different barriers regarding access to temperature assessment. This is attributed to the fact that there are various levels of knowledge, awareness, experience in diagnosis, socioeconomic status, geographic restrictions, and equipment available. This suggests that there is not “one-thermometer-fits-all” solution to the challenges faced in the health care context of low-resource settings like those in Uganda.

#### The Different Purposes of a Thermometer

Fever diagnostics plays an important role in monitoring fever-related illnesses as well as in reverse diagnostics (ie, to confirm or discard the suspicion of disease). The availability of diagnostic confirmation at home may increase willingness to receive treatment for fever from formal health care providers and reduce the morbidity and mortality rate caused by the delay of care. The first decision of treatment at home or in the community is especially important within the context of a restrictive community where people feel pressure from others in their social network when seeking care for febrile symptoms. As such, a thermometer that is designed for the purpose of reverse diagnostics or confirmation of fever in a household should have different properties than a thermometer designed for a clinical environment. For instance, the common digital fever thermometer may be expected to be easy to use, but in fact it requires literacy and a technological mental model to be used. In a clinical setting, hygiene, complementarity with other medical devices, size, and power lifetime are very important requirements [85-87]. However, whereas the focus given to accuracy and speed might make sense in a clinical environment, it does not make sense in a household environment since, in this case, the outcome-related decision is not clinical but, simply put, is represented by the question “Should I consult professional health care?” In the latter case, an easy interface design can help users distinguish severe from nonsevere illnesses by providing the states of body temperature with variations of visual interaction (eg, color and symbolic value) and auditory feedback rather than reporting a numeric value. This can be of importance since 43.5% of people who suffer from febrile symptoms treat their febrile illnesses by themselves at home. It is not desirable for all of these people to go to the doctor at the health centers and hospitals, since the workload at the health facilities is already too high [61]. In low-resource settings, costs are always crucial. As such, purchasing a thermometer instead of performing palpation, which is free, might be a barrier. However, a reliable fever indication by a thermometer could prevent overuse of medicines and unnecessary treatments and consequently reduce health care costs for the national health care system as well as for the patients themselves.

### Conclusions

This study presents an outline of the barriers of access to fever diagnostics in low-resource settings. This study also discusses an approach that may lead to an improved fever thermometer and help to reveal opportunities for innovative, complementary, and holistic initiatives to improve diagnostics of fever-related illnesses. On basis of the reviewed literature focused on sub-Saharan Africa, three complementary considerations were proposed that potentially have an impact in how fever diagnostics are designed and implemented in low-resource health care systems. Firstly, the fever diagnostics journey shows the involvement of people in the different phases of diagnostics, from awareness to monitoring and follow-up. Secondly, within the same health care system, there are different users of a fever thermometer for whom the conditions of access to fever diagnostics also differ. And thirdly, these different users have different needs regarding the information that is offered. The health care system in Uganda, as in other sub-Saharan countries, is greatly divided between public and private providers, and it is clear that the choices available for communities in low-resource settings are limited. In order to improve the overall access to fever diagnostics in these settings it is important to look into the specific and potential roles and needs that the different *users* may have. Needs related to fever diagnostics may include access to information about fever, information about its meaning and that of other illnesses, and clinical guidelines for handling and follow-up through appropriate channels. They may also include the need for appropriate thermometers and decision-making support. The involvement of health care professionals at all levels, community health workers, patients, and drug shop owners in a product-service system design approach may contribute to a more inclusive and holistic tackling of fever diagnostics.

The outcomes of this research are currently being used as direct input for the development of a new context-based product-service system for fever diagnostics in East Africa.

## Abbreviations

N/A: not applicable
